# Utility of the Social Vulnerability Index in Addressing Breast Cancer Disparities: A Meta‐Analysis

**DOI:** 10.1002/jso.70080

**Published:** 2025-09-20

**Authors:** Antoinette T. Nguyen, Rena A. Li, Nicole C. Ontiveros, Tarifa H. Adam, Nora Hansen, Robert D. Galiano

**Affiliations:** ^1^ University of Rochester School of Medicine and Dentistry Rochester USA; ^2^ Northwestern University Feinberg School of Medicine Chicago USA

**Keywords:** breast cancer, social determinants of health, Social Vulnerability index

## Abstract

**Objective:**

To evaluate the utility of the Social Vulnerability Index (SVI) in understanding disparities in breast cancer screening, incidence, and mortality.

**Background:**

Despite major advances in breast cancer detection and treatment, significant disparities persist—particularly among socioeconomically and geographically vulnerable populations. The SVI, developed by the CDC, is a composite index that captures community‐level vulnerability across multiple social domains and may serve as a tool to identify and address inequities in cancer care.

**Methods:**

This systematic review and meta‐analysis were conducted in accordance with PRISMA guidelines and registered in PROSPERO (CRD42024616874). PubMed, Scopus, and Embase were searched for studies examining associations between SVI and breast cancer outcomes. Studies were evaluated using the Newcastle‐Ottawa Scale or appropriate Cochrane tools. Meta‐analyses were performed where applicable.

**Results:**

Fifteen studies were included. Seven studies examined screening; a pooled meta‐analysis (*n* = 3) showed reduced screening in high‐SVI areas (pooled OR: 0.55, 95% CI: 0.24–1.26; I² = 99%). Four studies reported reduced incidence in high‐SVI populations, likely reflecting underdiagnosis. Five studies demonstrated increased mortality in high‐SVI populations, with ORs ranging from 1.09 to 2.84. Other studies addressed comorbidities, access to care, and disease subtypes.

**Conclusion:**

The SVI is a valuable, multidimensional tool for characterizing and addressing disparities in breast cancer outcomes, with implications for public health interventions and policy.

## 1 Introduction

Breast cancer remains a leading cause of cancer‐related morbidity and mortality worldwide, with over two million new cases diagnosed annually and over 685,000 deaths reported each year [1]. As one of the most extensively studied malignancies, advancements in breast cancer detection and treatment have significantly improved outcomes, yet disparities in care and survival persist [2]. These inequities are particularly pronounced among socioeconomically disadvantaged populations, racial and ethnic minorities, and individuals residing in regions with limited healthcare access [3, 4, 5, 6, 7]. Despite decades of public health campaigns aimed at promoting universal screening and early detection, gaps persist in screening participation rates and in timely diagnostic follow‐up after abnormal mammography findings.

Historically, marginalized populations have faced barriers to timely and equitable care. These barriers encompass financial hardship, lack of insurance, geographic isolation, limited health literacy, and systemic racism embedded in healthcare systems. For example, although African American women report similar rates of mammography use, they are more likely to experience diagnostic delays and late‐stage presentation, suggesting that screening access does not equate to timely and effective care [4, 8, 9]. Additionally, Hispanic and low‐income women often encounter delays in treatment initiation due to challenges in navigating healthcare systems and securing resources, which can lead to worse outcomes [3, 4, 10, 11]. These disparities are further exacerbated by historical underinvestment in healthcare infrastructure in marginalized communities and structural inequities, including redlining—a discriminatory housing policy that denied minority neighborhoods access to mortgages and investment—which continue to shape access to care. Addressing these challenges requires a nuanced understanding of how social, economic, and environmental factors shape disparities in the continuum of breast cancer care.

In recent years, the Social Vulnerability Index (SVI) has emerged as a promising tool for assessing the role of social determinants of health in healthcare disparities [12]. Developed by the Centers for Disease Control and Prevention (CDC), the SVI provides a comprehensive, multidimensional measure of a community's vulnerability to external stressors, integrating data across four domains: socioeconomic status, household composition, minority status and language, and housing and transportation [8]. By aggregating 15 key social indicators, the SVI generates a composite score that allows researchers and policymakers to stratify communities based on their capacity to withstand stressors, whether from natural disasters, public health crises, or systemic inequities in healthcare delivery [13]. Although originally designed to guide disaster response efforts, the SVI has been adapted to evaluate health disparities across numerous medical conditions, including cancer [14, 15, 16].

Breast cancer outcomes, encompassing screening participation, incidence rates, and mortality, are particularly sensitive to social vulnerabilities [17, 18, 19]. Higher SVI scores have been associated with reduced adherence to screening guidelines, delayed diagnoses, and increased mortality [10]. Integrating SVI into analyses of breast cancer outcomes can elucidate the complex interplay between social factors and clinical metrics, offering a lens through which to target interventions and resources more equitably [20, 21]. This review is the first to comprehensively synthesize breast cancer–specific SVI literature across screening, incidence, and mortality domains. Distinguishing itself from prior work, it critically evaluates methodological rigor, provides pooled effect estimates where appropriate, and identifies opportunities for clinical and policy action informed by SVI‐based stratification.

## 2 Methods

This systematic review and meta‐analysis followed PRISMA guidelines and was registered in PROSPERO (CRD42024616874). A comprehensive search was conducted in PubMed, Scopus, and Embase to identify studies assessing the relationship between the SVI and breast cancer outcomes (screening, incidence, and mortality) from database inception to January 10, 2025. The search strategy combined Medical Subject Headings (MeSH) and keyword terms using Boolean operators: (“breast cancer“ OR “breast carcinoma“ OR “breast neoplasm“) AND (“Social Vulnerability Index“ OR “SVI“ OR “social determinants of health“). Studies were limited to English‐language publications, and duplicates were removed. Rayyan was used to organize the studies.

**Figure 1:**
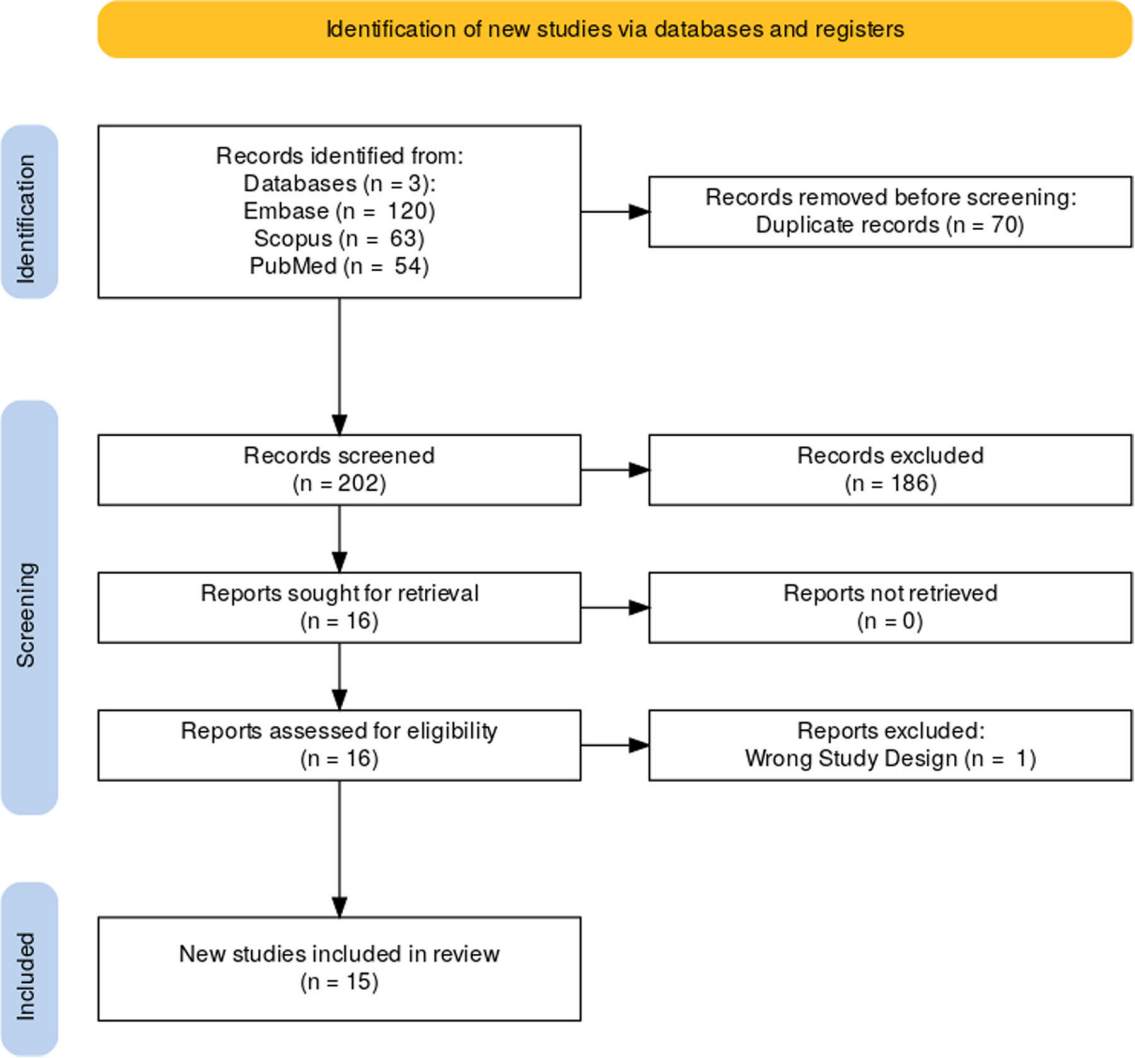
PRISMA Diagram. Flow diagram of study selection according to PRISMA guidelines.

Eligibility criteria included studies that reported quantitative associations between SVI and breast cancer outcomes, using odds ratios (ORs), hazard ratios (HRs), or relative risks (RRs). Studies were excluded if they: (1) focused on cancers other than breast cancer, (2) lacked SVI as an exposure variable, or (3) review articles, editorials, case reports, or conference abstracts without sufficient methodological detail. Screening and data extraction were conducted independently by two reviewers, with disagreements resolved by a third. Of 237 studies identified across PubMed, Scopus, and Embase, 202 unique records were screened, and 15 studies met inclusion criteria after full‐text review. Extracted data included study characteristics, SVI details, and effect measures. The study selection process is outlined in the PRISMA flow diagram (Figure 1). The Newcastle‐Ottawa Scale (NOS) was used for retrospective cohort studies, while the AXIS Tool was applied to cross‐sectional studies. The NIH Quality Assessment Tool for Observational Studies was used for ecological and geospatial analyses, and ROBINS‐I was used for quasi‐experimental studies. (Table 1) [22, 23]. Each study was independently assessed by two reviewers, and disagreements were resolved through consensus. The overall risk of bias for each study was categorized as low, moderate, or high based on aggregated scores.

**Table 1:** Risk of Bias of Included Studies.

Study	Study design	ROB tool used	Selection bias	Confounding bias	Outcome measurement	Data completeness	Overall risk
Mehta et al. 2024	Cross‐sectional database study	AXIS Tool	Moderate	Moderate	Moderate	Moderate	Moderate
Ashad‐Bishop et al. 2023	Ecological study	NIH Quality Assessment Tool for Observational Studies	Moderate	Moderate	Moderate	Moderate	Moderate
Davis et al. 2023	Ecological and machine learning	NIH Quality Assessment Tool for Observational Studies	High	High	Moderate	Moderate	Moderate
Phipps et al. 2024	Retrospective cohort study	Newcastle‐Ottawa Scale (NOS)	Low	Moderate	High	Moderate	High
Bauer et al. 2022	Cross‐sectional study	AXIS Tool	Moderate	Moderate	Moderate	Moderate	Moderate
Moazzam et al. 2023	Ecological study	NIH Quality Assessment Tool for Observational Studies	Moderate	Moderate	High	Moderate	High
Hwang et al. 2024	Ecological cross‐sectional study	AXIS Tool	Moderate	Moderate	Moderate	Moderate	Moderate
Hashtarkhani et al. 2024	Geospatial and machine learning	NIH Quality Assessment Tool for Observational Studies	Moderate	Moderate	High	Moderate	High
Hirko et al. 2024	Retrospective cohort study	Newcastle‐Ottawa Scale (NOS)	Low	Moderate	High	Moderate	High
Councell et al. 2024	Retrospective cohort study	Newcastle‐Ottawa Scale (NOS)	Low	Moderate	High	High	High
Aijazuddin et al. 2023	Cross‐sectional ecological study	AXIS Tool	Moderate	Moderate	Moderate	Moderate	Moderate
Mar et al. 2023	Geospatial and ecological analysis	NIH Quality Assessment Tool for Observational Studies	Moderate	Moderate	High	Moderate	High
Li et al. 2024	DID analysis	ROBINS‐I	Low	Low	High	High	High
Goldfinger et al. 2024	Spatiotemporal epidemiological	NIH Quality Assessment Tool for Observational Studies	Moderate	High	Moderate	Moderate	High
Chen et al. 2023	Cross‐sectional analysis	AXIS Tool	Moderate	Moderate	Moderate	Moderate	Moderate

Summary of risk of bias domains for all studies using appropriate tools based on study design.

Where applicable, a random‐effects meta‐analysis was performed to pool effect estimates, with heterogeneity assessed via the I² statistic. Only three studies met inclusion criteria for meta‐analysis of screening outcomes, due to the requirement for reporting odds ratios with 95% confidence intervals for comparable high‐ versus low‐SVI groups. Studies reporting risk ratios, hazard ratios, or descriptive statistics alone were excluded from pooling to maintain analytic consistency. Publication bias was evaluated using funnel plots. All analyses were conducted in R (4.4.2).

## 3 Results

**Figure 2:**
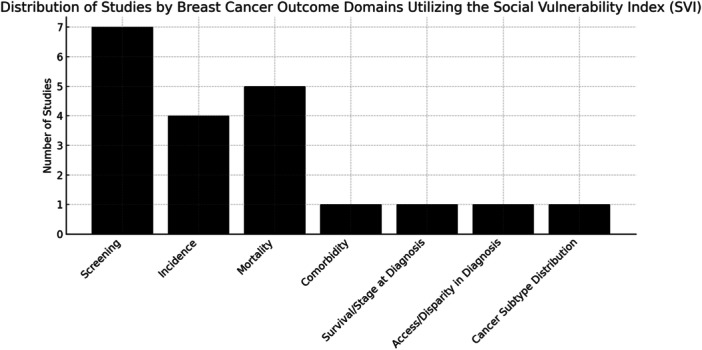
Distribution of Studies by Breast Cancer Outcome Domains Utilizing the Social Vulnerability Index (SVI). Bar chart illustrating the number of studies evaluating screening, incidence, mortality, and related outcomes using the SVI.

A total of 15 studies were included in this systematic review and meta‐analysis, addressing the relationship between the SVI and breast cancer outcomes. These studies, which encompassed breast cancer screening, incidence, and mortality, as well as other related outcomes, illustrate the significant impact of social and structural vulnerabilities on disparities in breast cancer care (Table 2, Figure 2).

**Table 2:** Summary of Included Studies.

Study	Study design	Methods	# of participants	Metric	Average SVI	Statistics and key findings	How SVI was used	Limitations
Mehta et al. 2024	Cross‐sectional database study	Logistic regression and ANOVA to compare SVI tertiles and cancer screening, incidence, and mortality rates	3132 U.S. counties	Breast cancer screening, incidence, and mortality	Calculated for each county (ranged from 0 to 1)	ORs for SVI's impact on screening and mortality, *p* < 0.001. High SVI correlated with lower screening rates and higher mortality for breast cancer; incidence lower in high SVI areas	SVI tertiles compared for screening, incidence, mortality metrics; adjusted for age	Cross‐sectional limits causal inference, county‐level aggregate limits individual‐level analysis
Ashad‐Bishop et al. 2023	Ecological study	Census tract‐level data analysis and cluster analysis	492 census tracts in Miami‐Dade County	Breast cancer screening (mammo‐graphy participation)	SVI calculated for Miami‐Dade, adapted with local determinants	ANOVA, Kruskal–Wallis test, *p* < 0.001. Lower screening rates in more vulnerable clusters, with breast cancer screening below county average in the most vulnerable areas	SVI clusters created from census data to assess screening disparity	Ecological limits individual‐level analysis; census tract estimates subject to misestimation
Davis et al. 2023	Ecological and machine learning study	Combined county‐level screening and age‐adjusted mortality data with CDC SVI to predict screening uptake, then applied model to patient‐level EMRs	2270 U.S. counties; 1880 patients at MUSC	Breast cancer screening, age‐adjusted mortality	Calculated per county (based on CDC data)	ROC AUC 0.532 (county); *p* < 0.001, with R² for minority areas 0.001. SVI predicts screening rates at county level but fails for individual prediction; screening‐mortality association weak in high minority areas	SVI used to develop county‐level patterns, but individual‐level predictions lacked accuracy	Differences between county‐ and individual‐level data, ecological fallacy, limited patient cohort
Phipps et al. 2024	Retrospec‐tive cohort study	Comparison of women in high‐risk screening program (HRSP) with those diagnosed with breast cancer; SVI evaluated by ZIP code	233 HRSP participants, 685 breast cancer diagnoses	Breast cancer screening and diagnosis	Median SVI 0.64 vs. 0.66 (*p* = 0.11)	Wilcoxon, Chi‐square tests; *p* < 0.05. HRSP participants were younger, more often White, with higher income and insurance coverage, highlighting screening disparities	SVI used to compare area‐based socioeconomic factors among groups	Limited generalizability beyond IU Simon Cancer Center; age limitation of ≤ 65
Bauer et al. 2022	Cross‐sectional study	Multilevel regression and Bayesian mixed‐effects models on county‐level screening data	3141 US counties	Breast cancer screening (USPSTF guideline adherence)	0.86 OR for highest versus lowest quintile (Q5 vs. Q1) for breast cancer screening	Adjusted ORs with 95% CrI (Q5 vs. Q1) 0.92 (95% CrI, 0.90–0.93). Higher SVI correlates with lower odds of screening adherence across quintiles; higher disparities in rural areas	SVI quintiles used to stratify counties by vulnerability and assess disparities in screening rates	Aggregate data limits individual‐level insights; ecological study design limits causal inference
Moazzam et al. 2023	Cross‐sectional, ecological analysis	Mixed‐effects logistic regression and mediation analysis on CDC SVI and cancer screening data	11,831 census tracts	Cancer screening rates for breast, colon, and cervical cancers	SVI based on CDC PLACES data	OR for screening targets in redlined tracts: breast 0.76, colon 0.34, cervical 0.21. Redlined areas less likely to meet cancer screening targets, with SVI mediating these effects	SVI used to adjust for socioeconomic and access‐to‐care disparities in redlined areas	Limited by ecological study design; individual‐level data not available
Hwang et al. 2024	Ecological cross‐sectional study	Used spatial autoregressive Tobit models with HealthFacts RI claims data	Over 1 million Rhode Island residents	Screening rates for breast cancer	SVI calculated per ZIP code tabulation area	β = −0.07 for each 1‐unit increase in SVI. Higher SVI, especially in socioeconomic domains, correlates with lower screening rates; specific SVI domains showed varying effects on different cancers	SVI domains analyzed for effects on screening rates at community level	Does not include uninsured populations; ZCTA‐level data limits individual prediction
Hashtarkhani et al. 2024	Geospatial and machine learning analysis	Analysis of spatial clusters using Getis‐Ord Gi* in ArcGIS; Random Forest model in R with RF variable importance ranking	12,409 breast cancer patients (2014–2021)	Charlson Comorbidity Index (CCI) and breast cancer incidence	SVI compared across Shelby County	RF model variable importance; RMSE minimization. SVI and racial segregation are major comorbidity predictors; hotspots align with high Black population tracts	SVI overlaid with hotspots to assess correlation with comorbidity clusters	Limited to Shelby County; ecological fallacy possible in spatial analysis
Hirko et al. 2024	Retrospec‐tive cohort study	Hierarchical logistic regression and Cox proportional hazards models; Kaplan‐Meier and log‐rank tests	586 women with IBC (1986–2021)	Stage at diagnosis and overall survival	County‐level SVI ranged from 2.6% to 95.8%	*p* = 0.63 for OS across SVI quartiles; OR (95% CI) = 0.99 (0.84, 1.16) for metastatic disease presence. SVI not associated with metastatic disease or OS; high‐quality care may mitigate socioeconomic factors	SVI linked with zip codes to analyze stage at diagnosis and survival rates	Limited to patients from one institution; findings may not generalize to broader populations
Councell et al. 2024	Retrospec‐tive cohort study	Cox regression analysis of all‐cause mortality by SVI quartiles	4843 breast cancer patients (2012–2022)	All‐cause mortality stratified by stage	SVI quartiles, national‐level 2018	Increased mortality in top SVI quartiles (65% and 90% higher). Higher SVI associated with greater mortality in Stages 1–3, but not in Stages 0 or 4	SVI linked with patient home address to assess mortality disparity	Limited to one institution; external validity may be limited
Aijazuddin et al. 2023	Cross‐sectional ecological study	Analysis of actual versus expected cancer incidence by SVI percentiles at the Census Tract level in NY State	4647 Census Tracts (97.71% of NY State's 2016 population)	Cancer incidence, including breast cancer	SVI percentile based on Census Tract	R = −0.90 for breast cancer; *p* < 0.001. Higher SVI associated with decreased breast cancer incidence, potentially due to lower screening rates	SVI used to identify micro‐geographic variations in cancer incidence and disparities	Limited to one state; Census Tract data may not capture individual‐level effects
Mar et al. 2023	Geospatial and ecological analysis	Created index using SVI and CDC data on cancer screening needs by Census Tract	1184 Census Tracts in PA and NJ	Priority index for cancer screening	Range 0.000000 to 0.938343	Mean index score 0.400593 (SD = 0.198556). Highest priority tracts in Philadelphia County, showing need for targeted screening	SVI incorporated into index with other social determinants to rank priority for MSU deployment	Limited to geographic and census tract data; validation needed
Li et al. 2024	Quasi‐experi‐mental, Difference‐in‐Difference (DID)	Analyzed SynCan data with Poisson regression and DID model across SVI tertiles	8,007 Census Tracts in California	Breast cancer incidence (overall and by stage)	SVI tertiles (high, medium, low)	Relative IRR = 1.03 (95% CI 1.00–1.06, *p* = 0.026) for high versus low SVI. Medicaid expansion had a significant effect on localized cancer incidence in high‐SVI neighborhoods	SVI was used to stratify census tracts for DID analysis to compare changes pre/post expansion	Limited to one state; potential influence of other ACA policies
Goldfinger et al. 2024	Spatio‐temporal epidemio‐logical analysis	Spatial and space‐time clustering analysis; spatial regression	935 TNBC cases across 518 tracts and 96 neighbor‐hoods	TNBC prevalence rates by census tract	Mean SVI of 0.64 for tracts and 0.63 for neighbor‐hoods	Brownfield areas associated with TNBC at tract‐level (β = 4.27, *p* < 0.001) and neighborhood‐level (β = 8.61, *p* < 0.001). High TNBC clusters in disadvantaged non‐Hispanic Black neighborhoods; brownfields significant TNBC correlate	SVI initially considered as a predictor but removed due to collinearity with education	Limited generalizability outside Miami‐Dade; results not validated in a broader population
Chen et al. 2023	Cross‐sectional analysis	Linked CDC WONDER age‐adjusted mortality rates with county‐level SVI data; robust linear regression	3143 US counties	Cancer‐related mortality	SVI quartiles by county	RR 1.09 (95% CI [1.08, 1.10]) for highest versus lowest SVI. Higher mortality in high‐SVI counties, especially in rural and Southern regions; most pronounced for lung and colorectal cancers	SVI used to stratify counties for mortality comparisons	Limited to county‐level data; potential confounding by unmeasured individual factors

Summary of study design, methodology, population characteristics, SVI use, key findings, and limitations.

Seven studies examined the association between SVI and breast cancer screening, consistently demonstrating that higher SVI correlates with reduced screening participation. Mehta et al. (2024) reported that counties in the highest SVI tertile had significantly lower odds of meeting the U.S. median screening rate (OR 0.24, 95% CI: 0.20–0.29) compared to counties in the lowest tertile [24]. Similarly, Bauer et al. (2022) observed that counties in the highest SVI quintile showed reduced adherence to screening guidelines compared to those in the lowest SVI quintile (OR 0.92, 95% CI: 0.90–0.93) [25]. Moazzam et al. (2023) highlighted the impact of historical redlining, with an OR of 0.76 (95% CI: 0.64–0.91) for achieving screening targets in redlined areas compared to less vulnerable tracts [26]. Ashad‐Bishop et al. (2024) analyzed mammography participation in Miami‐Dade County and found significantly lower rates in high‐SVI census tracts compared to county averages, emphasizing hyperlocal disparities [27]. Davis et al. (2023) demonstrated that while SVI predicted county‐level mammogram uptake, it failed to translate to accurate individual‐level predictions, particularly in areas with high minority populations [28]. Additional geospatial studies, including those by Hwang et al. (2024) and Mar et al. (2023), further corroborated these findings, with high‐SVI tracts consistently underperforming in screening participation [29, 30].

**Figure 3:**
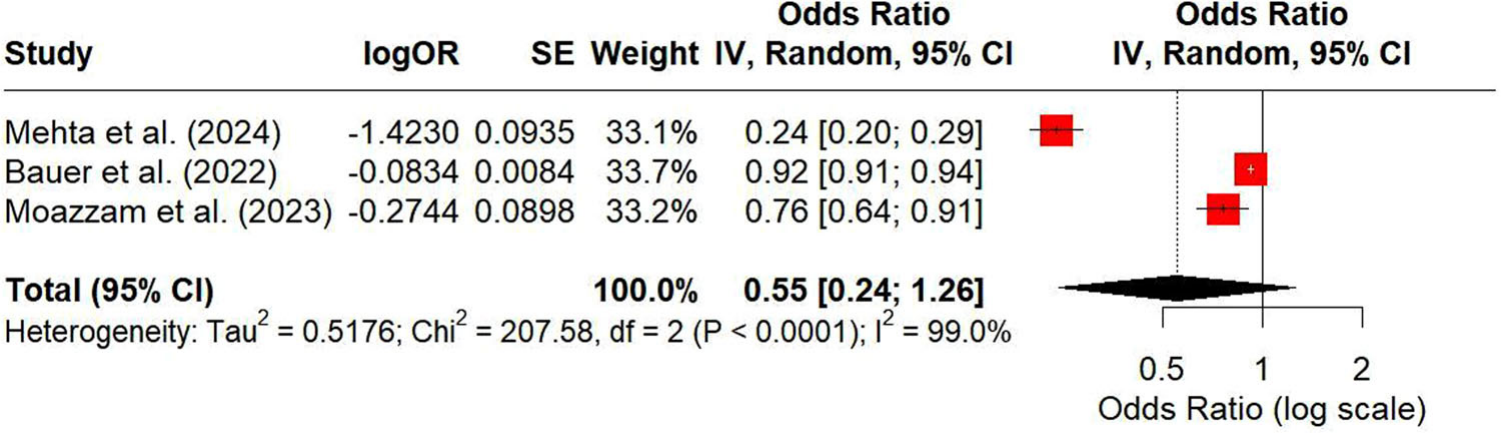
Social Vulnerability Index (SVI) and Breast Cancer Screening Outcomes Meta‐Analysis Forest Plot. Forest plot showing pooled odds ratios for breast cancer screening rates in high‐SVI versus low‐SVI populations.

**Figure 4:**
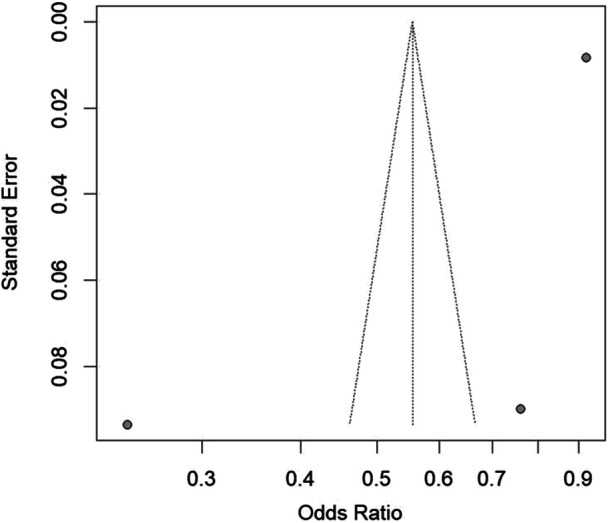
Social Vulnerability Index (SVI) and Breast Cancer Screening Outcomes Meta‐Analysis Funnel Plot. Funnel plot assessing potential publication bias among studies included in the meta‐analysis.

A meta‐analysis was conducted to evaluate the impact of SVI on breast cancer screening outcomes. Three studies were included: Mehta et al. (2024), Bauer et al. (2022), and Moazzam et al. (2023) [23, 24, 25]. The pooled analysis yielded a combined OR of 0.55 (95% CI: 0.24–1.26), suggesting that individuals in high‐SVI areas are approximately 45% less likely to meet breast cancer screening targets compared to those in low‐SVI areas. However, the wide confidence interval crossing 1.00 indicates that the pooled effect is not statistically significant. Of note, heterogeneity across the studies was substantial (I² = 99%), reflecting significant differences in study populations, methodologies, or other factors (Figures 3, 4).

Four studies addressed breast cancer incidence and found that reduced screening in high‐SVI areas led to lower reported incidence, likely masking the true burden of disease. Mehta et al. (2024) reported that counties in the highest SVI tertile had significantly lower odds of exceeding the U.S. median incidence rate (OR 0.33, 95% CI: 0.27–0.40) [24]. Aijazuddin et al. (2023) observed a strong inverse correlation between SVI and incidence (R = −0.90, *p* < 0.001), further bolstering the connection between reduced screening and underreported cases [31]. Li et al. (2024) examined the impact of Medicaid expansion, demonstrating that localized cancer incidence increased in high‐SVI neighborhoods following expansion, highlighting the potential for policy interventions to mitigate disparities [32]. Finally, Mar et al. (2023) used geospatial analyses to identify high‐priority neighborhoods for intervention, demonstrating that tracts with higher SVI scores consistently fell below median incidence rates, highlighting barriers to care and detection. Although some populations—such as Hispanic and Asian women—have lower overall incidence rates, the studies included in this review primarily attributed reduced incidence in high‐SVI areas to limited access to screening, rather than biological differences alone. Further studies are warranted to parse these overlapping effects.

Mortality outcomes were explored in five studies, and consistently showed higher mortality rates in populations with greater social vulnerability [33]. Mehta et al. (2024) reported an OR of 2.84 (95% CI: 2.22–3.62) for breast cancer mortality in the highest SVI tertile compared to the lowest [24]. Councell et al. (2024) found that patients in the highest SVI quartile had a 90% higher risk of all‐cause mortality (HR 1.90, *p* < 0.01) compared to those in the lowest quartile after adjusting for age and breast cancer stage at diagnosis [19]. Chen et al. (2023) reported that counties in the highest SVI quartile had significantly higher cancer‐related mortality (RR 1.09, 95% CI: 1.08–1.10) [34]. Ashad‐Bishop et al. (2024) highlighted disparities in age‐adjusted mortality rates in Miami‐Dade County, noting that high‐SVI clusters experienced disproportionately higher risks of mortality [27]. Hirko et al. (2024) corroborate these findings of higher SVI scores correlating to higher mortality rates [33].

Beyond the aforementioned primary outcomes, several studies explored the SVI's association to related themes, including comorbidities, geospatial disparities, access to care, and the effects of systemic inequities. For example, Hashtarkhani et al. (2024) demonstrated that both the SVI and racial segregation were significant predictors of breast cancer comorbidities, with higher Charlson Comorbidity Index scores clustering in areas with elevated SVI [35]. Similarly, Goldfinger et al. (2024) reported a higher prevalence of triple‐negative breast cancer in high‐SVI neighborhoods, underscoring the intersection of aggressive disease subtypes and social vulnerability [36]. While triple‐negative breast cancer (TNBC) prevalence is well‐established among Black women, Goldfinger et al. (2024) controlled for racial composition and still identified an independent association between high SVI and TNBC clusters, suggesting that structural and environmental stressors may compound biologic susceptibility. Phipps et al. (2024) further highlighted disparities by comparing individuals in high‐risk screening programs to those diagnosed with breast cancer, revealing that younger, wealthier, and insured individuals were more likely to access proactive care [37]. In response to these disparities, Mar et al. (2023) developed a geospatial priority index for mobile screening units, emphasizing the importance of targeted interventions in vulnerable communities [30]. Additionally, Moazzam et al. (2023) and Li et al. (2024) highlighted the role of policy solutions, such as Medicaid expansion and equitable resource allocation, in addressing systemic inequities [26, 32].

## 4 Discussion

This systematic review and meta‐analysis offers a comprehensive synthesis of the association between the SVI and breast cancer outcomes, including screening, incidence, and mortality. The analysis of 15 studies highlights SVI as a pivotal measure for identifying disparities and informing targeted interventions. The results underscore significant inequities disproportionately impacting high‐SVI populations, with critical implications for clinical practice, public health policy, and resource allocation.

The SVI proved highly effective in capturing multidimensional aspects of vulnerability, including socioeconomic status, minority status, and access to resources [38, 39]. Its integration into breast cancer research enabled the quantification of disparities at individual, community, and regional levels. Studies such as Mehta et al. (2024) and Bauer et al. (2022) demonstrated the utility of SVI in identifying counties with reduced screening adherence, while Moazzam et al. (2023) and Ashad‐Bishop et al. (2024) highlighted the SVI's ability to localize disparities at finer scales, such as census tracts [24, 25, 26, 27]. Importantly, the index allowed researchers to identify hyperlocal trends that might otherwise be obscured by aggregate analyses, underscoring the measure's value in guiding resource allocation for screening programs, such as mobile mammography units or Medicaid expansion [40, 41]. However, the SVI also revealed significant heterogeneity in its application. Some studies, such as Davis et al. (2023), highlighted its limitations in predicting individual‐level outcomes, emphasizing the need to complement SVI analyses with patient‐level data [28]. Moreover, while most studies focused on screening and mortality, fewer addressed the intermediate step of incidence, which remains underexplored despite its critical role in understanding the continuum of breast cancer care.

In addition, the findings consistently demonstrated that higher SVI scores are associated with lower odds of meeting breast cancer screening targets. The pooled meta‐analysis for screening outcomes revealed a 45% reduction in the likelihood of high‐SVI populations adhering to screening guidelines compared to low‐SVI populations. The I² statistic of 99% indicates substantial heterogeneity, likely reflecting differences in geographic scope, SVI domain emphasis, and screening benchmarks. This limits the generalizability of the pooled estimate. While only three studies were eligible for pooled analysis, this reflects both the early stage of SVI‐specific research and the variation in reported outcome metrics. Studies such as those by Mehta et al. (2024) and Bauer et al. (2022) provided robust quantitative data, while localized analyses like Ashad‐Bishop et al. (2024) offered insights into hyperlocal disparities, particularly in diverse urban areas [24, 25, 27]. The consistent association between high SVI and reduced screening rates emphasizes the need for tailored interventions, such as culturally sensitive outreach, community health education, and expanded access to mobile screening units [42, 43, 44].

Reduced incidence rates in high‐SVI populations, as highlighted by Mehta et al. (2024) and Aijazuddin et al. (2023), likely reflect the downstream effects of inadequate screening [24, 31]. These findings are consistent with known patterns in which delayed or absent screening leads to underreporting of cases until advanced stages of disease [45]. This underreporting not only obscures the true burden of breast cancer in vulnerable populations but also hampers the ability to allocate resources effectively [46, 47]. Li et al. (2024) demonstrated that policy changes, such as Medicaid expansion, could mitigate some of these disparities by increasing early diagnoses in high‐SVI areas [32]. However, further research is needed to disentangle the extent to which reduced screening contributes to lower reported incidence versus reflecting true differences in disease prevalence. Furthermore, high‐SVI populations consistently exhibited increased breast cancer mortality. Mehta et al. (2024) reported substantially higher mortality risks in the highest SVI tertiles and quartiles, even after adjusting for age and breast cancer stage at diagnosis. These findings were further corroborated by Chen et al. (2023) and Ashad‐Bishop et al. (2024), who highlighted the compounded impact of social vulnerabilities on mortality [19, 27]. The elevated mortality in high‐SVI populations underscores the need for systemic interventions beyond screening, including timely access to diagnostic services, equitable treatment options, and robust social support systems [48, 49, 50].

Several outcomes associated with high SVI may in part reflect racial composition, given that neighborhoods with high SVI often correlate with marginalized racial and ethnic groups. For example, triple‐negative breast cancer is more prevalent in Black women, raising the possibility that some findings attributed to social vulnerability may be partially mediated by race. Nevertheless, SVI captures a broader constellation of social risk factors beyond race, including transportation access, education, and housing quality.

These findings underscore the need to integrate SVI into health systems' decision‐making, including tailored resource allocation, implementation of mobile screening units in high‐SVI areas, and culturally responsive patient navigation programs. Furthermore, policies such as Medicaid expansion and targeted community partnerships could help bridge structural gaps.

### 4.1 Limitations

While the findings of this systematic review and meta‐analysis offer compelling evidence of the SVI's utility, several limitations of the included studies warrant consideration. Given the wide confidence interval and I² of 99%, the pooled estimate must be interpreted with caution. The heterogeneity likely reflects differences in population demographics, study design, and geographic context. Furthermore, given the limited number of studies (*n* = 3) included in the meta‐analysis, subgroup or sensitivity analyses were not feasible; however, future work should examine heterogeneity by study design, geography, and SVI component domains. Finally, while the SVI is a valuable tool, it is inherently limited by its ecological design and static nature. It captures community‐level vulnerability but may obscure important intra‐community variation and does not account for temporal dynamics such as population shifts or gentrification. Most included studies were cross‐sectional or ecological, limiting causal inference and individual‐level attribution. Future research should incorporate longitudinal designs and granular, patient‐level SVI applications to improve precision and policy relevance Future research should also aim to harmonize methodologies, expand geographic diversity, and incorporate longitudinal designs to better capture the dynamic interplay between social vulnerability and breast cancer outcomes.

## 5 Conclusion

This systematic review and meta‐analysis highlight the SVI as a valuable tool for understanding and addressing disparities in breast cancer outcomes, including screening, incidence, and mortality. Populations with high SVI consistently face lower screening rates, underreported incidence, and higher mortality – patterns driven by structural inequities that extend beyond individual clinical care. The findings reinforce the need for targeted public health interventions and strategic resource allocation informed by SVI. These insights emphasize the importance of integrating social vulnerability into patient care to ensure timely access to screening and treatment for those at greatest risk. Future research should aim to refine the application of SVI and develop interventions that directly address the systemic barriers to equitable breast cancer care.

## Synopsis

This systematic review and meta‐analysis examines how the Social Vulnerability Index (SVI) is used to assess disparities in breast cancer screening, incidence, and mortality. Synthesizing data from 15 studies, it highlights key sociodemographic factors and offers insight into how SVI‐driven approaches can inform equitable cancer care strategies.

## Supporting information

PRISMA checklist svi breast.

## Data Availability

The data that support the findings of this study are available in Google at https://www.google.com/. These data were derived from the following resources available in the public domain: ‐ PubMed, https://pubmed.ncbi.nlm.nih.gov/ ‐ Cochrane, https://www.cochrane.org/ ‐ Embase, https://www.embase.com/landing?status=yellow.
